# Supplementary material for: Usefulness of circulating microRNAs miR-146a and miR-16-5p as prognostic biomarkers in community-acquired pneumonia
Source: PLoS One. 2020 Oct 23;15(10):e0240926. doi: 10.1371/journal.pone.0240926 (PMC7584179; doi:10.1371/journal.pone.0240926)
Supplement: S1 Appendix — (DOCX) [file pone.0240926.s001.docx]

**S1 APPENDIX**

**S1 Table: Clinical manifestations, physical examination, radiology, and severity scales at admission**

**S2 Table: Blood test within first 24 hours of admission**

**S3 Table: Analysis of baseline differences between patients with valid samples for miRNAs determination and patients with invalid samples**

**S4 Table: Detectable miRNA levels of the 25 miRNAs of interest in valid selected patient samples.**

Table shows for each miRNA the number of patients with detectable miRNA and their percentage referred to total number of patients with valid samples in each group. miRNAs excluded due to incomplete data across patients with valid samples are highlighted in gray.**S1 Fig: Flow chart showing initial patient inclusion and subsequent exclusion according to quality control criteria of miRNA samples.**

**S2 Fig: Logistic regression equation of the multivariate models used in this work.**

380 patients hospitalized with diagnosis of CAP,
in H.U. de La Princesa Madrid, in 2015

**Exclusion during recruitment**

- Different main diagnosis.

- First blood test more than 24 hours after admission.

- Refusal to sign the Informed Consent.

153 patients included for descriptive study

117 samples of patients finally used for full analysis

**Exclusion during analysis**

Samples invalid according to quality technical criteria for miRNA analysis

| Quality process | Criteria | Samples that did not pass |
| --- | --- | --- |
| Hemolysis | miR-451 - miR-23a > 7 Cts | 6 |
| RNA extraction: differences between samples | UniSp2 < 2.5 Cts or > 2.5 Cts according to the median | 14 |
| RNA extraction: differences between samples | UniSp4 < 2,5 Cts or > 2,5 Cts according to the median | 2 |
| RNA extraction: differences between samples | UniSp5 > 38,86 Cts (2 SD) or undetectable | 8 |
| RNA extraction: differences within each sample | UniSp4 - UniSp 2 > 7 Cts  or < 5 Cts. | 5 |
| RNA extraction: differences within each sample | UniSp5 - UniSp 4 > 10 Cts  or < 5 Cts. | 1 |
| RT to cDNA: differences between samples | UniSp6 < 2.5 Cts o > 2.5 Cts according to the median | 0 |

**Logistic regression equation for adjusted multivariate model**


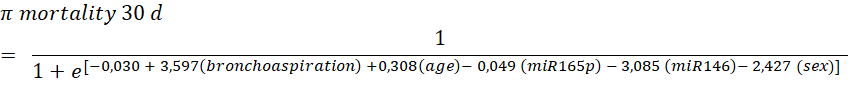


Where *bronchoaspiration* is worth 0 when it is absent and 1 when it is present; *age* is the age at admission minus 81.56; *miR165p* is the relative value of miR-16-5p minus 33.7; *miR146* is the relative value of miR-146a minus 0.336, and; *sex* is worth 0 in males and 1 in females.

**Logistic regression equation for non-adjusted multivariate model**


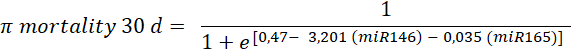


Where *miR146* is the relative value of miR-146a minus 0.336 and *miR165p* is the relative value of miR-16-5p minus 33.7.
