# Supplementary material for: Usefulness of circulating microRNAs miR-146a and miR-16-5p as prognostic biomarkers in community-acquired pneumonia
Source: PLoS One. 2020 Oct 23;15(10):e0240926. doi: 10.1371/journal.pone.0240926 (PMC7584179; doi:10.1371/journal.pone.0240926)
Supplement: Attachment — Submitted filename: Response to Reviewers.docx [file pone.0240926.s002.docx]

**PONE-D-20-09507
Usefulness of circulating microRNAs miR-146a and miR-16-5p as prognostic biomarkers in community-acquired pneumonia**

-----------

***Response to Reviewers***

All questions and suggestions raised by the reviewers are shown marked **in blue**, and our replies are marked **in black**, in order to facilitate a better understanding.

**Reviewer #1: Thank you for the opportunity to review the interesting manuscript of Galván-Román and colleagues. The study addresses an important topic of novel biomarkes for infections, and indeed miRs seem to be an appropriate target to enrich our current biomarker panels and prognostic score. One of the strengths of this study is the prospective and well-structured procedure of patient selection. Furthermore, the used methods for miR analyzation is adequate and clearly described.
In the following I listed my concerns that I would like to discuss with the authors.

Abstract:
The statement (CAP = inflammation = death) is in my opinion problematic. CAP often is associated with sepsis leading to death - thus, immunosuppression should also be considered as relevant risk factor. Therefore, I suggest rephrasing in “dysregulated” host response is more appropriate**.

**We agree with** **reviewer's suggestion and have rephrased the aforementioned statement as follows: "Patients with community-acquired pneumonia (CAP) undergo a dysregulated host response that is related to mortality"**

**Please provide at least some descriptive statistics (eg. mean/median) of the relative expression when comparing survivors and non-survivors. Also providen 95%-CI for the AUCs in the abstract.**

**Values of relative expression in survivors vs non-survivors as well as 95 CI for AUCs have been included in the revised abstract, as recommended.**

**Introduction:
Line 97: Improve “prognosis” or “prediction of prognosis”?**

**It is indeed a mistake. The sentence has been corrected including "improve of prediction of CAP prognosis"**

**Line 124: MiRNA are also widly used as biomarkes in infections (e.g. in sepsis) I would rather cite such studies instead of referring to malignancies or AID.**

**Line 125: This is not true. There are several studies that already have investigeated miRs in infections! Thus, the authors should summarize the current literature of miRs and infections more appropriately.**

**We have clarified this part of the introduction.**

**There are many studies investigating circulating miRNAs to diagnose both sepsis and a large number of specific infections. However, there are few studies that have tested the prognostic ability of circulating miRNAs specifically in community-acquired pneumonia. We wanted to stress this point as it highlights the importance of exploring the prognostic value of miRNAs in CAP. We have added text and references mentioning their utility in diagnosis, and we have slightly changed the original statement to clarify that when we state that there is limited literature, we are referring to their utility as prognostic markers.**

**The full paragraph would read as follows:**

**"The determination of miRNA in peripheral blood has been used for the diagnosis of malignancies, cardiovascular diseases or autoimmune disorders. [18] In addition, several studies have stablished the utility of circulating miRNAs in the diagnosis of sepsis** [*Circulating MicroRNAs as Biomarkers for Sepsis from Benz et al*.] **and in various specific infections (e.g. HIV, viral hepatitis, or tuberculosis)** [*Circulating MicroRNAs: Potential and Emerging Biomarkers for Diagnosis of Human Infectious Diseases from Verma et al.* ].

**However, there is limited scientific literature on the utility of these molecules as prognostic markers in CAP".**

**Methods:
On the one hand the authors write about a study of 752 miR using the miRCURY LNA panel. I think the authors should include this data in the supplements.**

**All these data have been included in a data repository within Zotero.org with URL: 10.5281/zenodo.3930832**

**Furthermore, 25 miRs are described in the methodology, whereas only 12 are presented in the results. Please provide also these at least as supplementary materials. Alternatively, the authors should mention that only part of the data was used for this work and the other data is evaluated separately. This should also be noted in the Data Availability statement.**

**The relative values of the 13 miRNAs not shown are of no interest for the present investigation since many patients did not present detectable values of these miRNAs. However, these results have been included in the repository, both as raw data and after normalization. In addition, a descriptive analysis is included showing the percentages of valid data for each miRNA in the annexes, as Supplementary Table 4.**

**This article is part of a research project started 5 years ago on biomarkers in CAP. The rest of the data, which includes blood parameters, and cardiovascular events at 30 days and 1 year of follow-up, can be found in other articles published by the group [21, 22]**

**Hence, the following paragraph has been added in Material and Methods:**

***This cohort has been previously used in other studies, in the context of a larger research project on prognostic biomarkers in CAP. However, all the data generated during this research is openly available in the public repository of Zenodo.org [DOI: 10.5281/zenodo.3930832]. Furthermore, the methodology followed can be found in the previously published protocol [23].***

**In addition, no miRs that were chosen as normalizers are presented, but their prognostic miRs were normalized against UniSP2 as derived from literature.**

**The raw results (Cts) and after linear transformation with the formula RQ = 2 ^ - (miRNA of interest - UniSP2) can be found in the supplemental data that have been uploaded to the aforementioned Zenodo.org repository.**

**Was UniSP2 also in their data set the “best” normalizer?**

**No, it was not, but there were no great differences in terms of coefficients of variation, either with mean or with median, which is expected considering that they are exogenous miRNAs added with the same concentration. The choice of UniSp2 for delta Ct was favoured because it is the most abundant exogenous miRNA and because it is a method commonly used in the literature** [Gómez-Dos-Santos V, Ramos-Muñoz E, García-Bermejo ML, et al. MicroRNAs in Kidney Machine Perfusion Fluid as Novel Biomarkers for Graft Function. Normalization Methods for miRNAs Profile Analysis. *Transplant Proc*. 2019;51(2):307-310. doi:10.1016/j.transproceed.2018.09.019].

**In any case, we carried out a double normalization, by exogenous miRNA (delta CT) and by a combination of endogenous miRNAs (NF (Normalizer Factor) calculated according to the geometric mean of RQ (Relative Quantity) of the 4 normalizers chosen per sample).**

**NRQ (normalized relative quantity) = RQ /NF**

Where,

RQ = 2^-(miRNA - UniSp2)

NF= geometric mean of RQs of miR-103a-3p, miR-23b-3p, miR-23a-3p and miR -25-3p

**All this information can be found in the supplementary data uploaded in the repository.**

**The authors should also provide some data describing UniSP2 as appropriate normalizer/reference (Mean with IQR + Coefficient of variation - when comparing survivors and non-survivors).**

**After comparing means (Cts) of the different spike-ins between survivors and non-survivors, we found no statistically significant differences, with an almost perfect correlation.**

| **Independent Samples Test** | | | | | | | | | | |
| --- | --- | --- | --- | --- | --- | --- | --- | --- | --- | --- |
|  |  | Levene's Test for Equality of Variances | | t-test for Equality of Means |  | Sig. (2-tailed) |  | Std. Error Difference | 95% Confidence Interval of the Difference | |
|  |  |  |  |  | df |  | Mean Difference |  |  |  |
|  |  | F | Sig. |  |  |  |  |  | Lower | Upper |
| miR-UniSp6 | Equal variances assumed | 0.216 | 0.643 | -0.695 | 115 | 0.488 | -0.23410 | 0.33674 | -0.90111 | 0.43291 |
|  | Equal variances not assumed |  |  | -0.702 | 12.227 | 0.496 | -0.23410 | 0.33339 | -0.95900 | 0.49080 |
| miR-UniSp2 | Equal variances assumed | 0.589 | 0.444 | 0.879 | 115 | 0.381 | 0.25795 | 0.29350 | -0.32342 | 0.83932 |
|  | Equal variances not assumed |  |  | 0.737 | 11.387 | 0.476 | 0.25795 | 0.35017 | -0.50958 | 1.02548 |
| miR-UniSp4 | Equal variances assumed | 0.418 | 0.519 | 1.101 | 115 | 0.273 | 0.34310 | 0.31174 | -0.27440 | 0.96060 |
|  | Equal variances not assumed |  |  | 0.972 | 11.585 | 0.351 | 0.34310 | 0.35287 | -0.42881 | 1.11500 |
| miR-UniSp5 | Equal variances assumed | 3.493 | 0.064 | 0.956 | 115 | 0.341 | 0.30248 | 0.31644 | -0.32433 | 0.92929 |
|  | Equal variances not assumed |  |  | 0.686 | 10.932 | 0.507 | 0.30248 | 0.44065 | -0.66813 | 1.27309 |

**We show this information to the reviewer; however, we believe that it may be of little interest to the reader, so we did not include it in the revised manuscript.**

**The authors should consider to correct their miR results for multiple testing.**

**We evaluated carrying out multiple testing like Bonferroni or FDR for the selection of the statistically significant miRNAs. We discarded that approach to avoid falling into a type I error, given this type of error would have an impact on a project, like ours, searching for potential biomarkers among a huge number of candidates. We would rather fall into a type II error, which would be later minimized or nullified with the multivariate regression model carried out afterwards. With this approach we expected missing as few potentially interesting candidates as possible.**

**Unfortunately, I am not able to follow the C-statics to the unadjusted and adjusted model. How are the underlying values/measures for these models composed in detail.**

**Indeed, it may be a complex analysis since there are multiple potential confounding clinical variables in addition to the two miRNAs with potential prognostic power. Therefore, the statistical complexity and the huge amount of results meant that in many cases not all the intermediate processes were shown.**

**Briefly, a univariate analysis was first performed for both the miRNAs of interest in this study and the possible confounding factors already described in the literature as associated with mortality in CAP patients.**

**With this analysis, the individual effect on 30-day mortality was assessed for each of the variables. Subsequently, we carried out a multivariate analysis to assess the independent effect of the statistically significant miRNAs (p≤0.05) from the univariate analysis (Table 2). To assess the contribution of potential confounding factors, variables with p ≤ 0.10 in the univariate analysis were chosen (*hierarchical model*, Table 1 and Supplementary table 1 and 2) as well as variables described as potentially significant in all previous published studies in CAP patients. (e.g. sex, *forced regression model*).**

**This created a multivariate model that was statistically valid (saturated model). However, we decided to progressively optimize the model until obtaining a model with a reduced number of variables (*parsimonious model*), but with a good goodness of fit, measured through the likelihood ratio (shown in Table 3).**

**With this final multivariate model (*parsimonious model*) adjusted by the confounding variables created with the binary logistic regression model, we estimated the weights of miR-16-5p and miR-146a, and predicted the probability of death at 30 days (Pr (ŷ=1)), obtaining a ROC curve (Figure 3).**

**This ROC curve was elaborated with the probability estimated by the parsimonious model, taking into account all the variables used to generate the model. Therefore the direct clinical interpretation of a cut-off point is not possible. On the other hand, it does allow comparison with other prognostic scores, by comparing AUCs. These considerations, together with the fact that miRNAs are measured in relative quantities, explain why an optimal cut-off point is not provided.**

**Were new sub-scores created?**

**There are several reasons why we did not want to display a sub-score. Most importantly, a forecast scale obtained through a multivariate model must be validated before it can be formally proposed. In addition, as we have previously mentioned, the absolute value of each miRNA can vary greatly according to the normalization applied; hence, unless this issue is improved, we believe that a sub-score should not be generalized as a prognostic score.**

**However, and in response to the reviewer's request, we can show a simulation of a sub-score consisting of the miR-16-5p and CURB-65. The resulting AUC would be 0.75, not much better than that calculated using only CURB-65 in our sample (0.72).**

```
Logistic regression                             Number of obs     =         95
                                                LR chi2(4)        =       9.74
                                                Prob > chi2       =     0.0450
Log likelihood = -29.182627                     Pseudo R2         =     0.1430

--------------------------------------------------------------------------------
    EXITUS_30D | Odds Ratio   Std. Err.      z    P>|z|     [95% Conf. Interval]
---------------+----------------------------------------------------------------
hsamiR165p_NUM |   .9808377   .0135127    -1.40   0.160     .9547077    1.007683
               |
        curb65 |
            1  |          1  (empty)
            2  |   .0201916   .0324289    -2.43   0.015     .0008671    .4701651
            3  |   .0899467   .1193442    -1.82   0.069      .006677    1.211678
            4  |   .0741205   .1023513    -1.88   0.060     .0049492    1.110053
            5  |          1  (omitted)
               |
         _cons |   3.947329    5.24316     1.03   0.301     .2921925    53.32583
--------------------------------------------------------------------------------
```

**How were the individual model parameters weighted? It must be clear for the reader what this “value” is that is applied in the ROC analysis. The authors should therefore explain this in much more detail, especially because the adjusted model has a very impressive AUC, which is significantly higher than the normal AUC.**

**They were not weighted; they are those obtained from the most parsimonious model using the following equation**

**Where bronchoaspiration value is 0 when it is absent and 1 when it is present; age is the age at admission minus 81.56; miR165p is the relative value of miR-16-5p minus 33.7; R146 is the relative value of miR-146a minus 0.336, and sex is 0 for males and 1 for females.**

**A similar equation was generated for the non-adjusted multivariate model**

**where *miR146* is the relative value of miR-146a minus 0.336 and *miR165p* is the relative value of miR-16-5p minus 33.7.**

**Both equations have been incorporated as Supplementary Figure 2 in the annex section.**

**Regarding to the AUC value, the true prognostic value of our model should be validated in other scenarios, adjusting and simplifying the model until it becomes manageable from a clinical point of view. On the other hand, it is not surprising that a simplified model (with categorized values) such as CURB-65 or PSI does not achieve such a high AUC in a specific sample of patients with CAP.**

**In any case, the objective of the comparison was not to suggest that our model could be superior to widely validated and widely used prognostic scales. Rather, it was to argue that miRNAs may also be used, in the future, within prognostic scores, probably translating inflammatory factors that may not be not entirely contemplated on traditional scales.**

**Furthermore, I suggest that the authors should consider any form of reclassification statistics to substantially show a prognostic benefit, e.g. by implementing one of the miRs (or both) in the LIS or CURB-65.**

**We have shown above an example using miR-16-5p and CURB-65. We believe that such information should not be incorporated into the manuscript, since AUC for this model is not significantly better that AUC for CURB-65.**

**I think that these study patients were also used in different studies (e.g. citation 37-39?). If so, this need to be clarified in the methods.**

**We agree with this comment. The following paragraph has been added at the end of the first part of Material and Methods:**

***This cohort has been previously used in other studies, in the context of a larger research project on prognostic biomarkers in CAP. All the data generated in this research are openly available in the public repository of Zenodo.org [DOI: 10.5281/zenodo.3930832]. Furthermore, the methodology followed can be found in the previously published protocol [21].***

**Results:
Is there any association with disease severity (e.g. when comparing ICU and non-ICU patients, or correlation with LIS?).**

**We believe that it would have been very interesting to analyse other outcome variables, such as admission to the ICU or other CAP complications. However, we thought that this analysis would add complexity to the results presented in the manuscript.**

**Furthermore, with respect to ICU, only two patients were admitted to the ICU during their hospitalization for CAP, therefore it would not be possible to determine whether miRNA levels were associated with this complication.**

**Reviewer #2:** **In their study “Usefulness of circulating miR-146a and miR-16-5p microRNAs as prognostic biomarkers in community-acquired pneumonia”, the authors investigate the potential of a panel of plasma microRNAs to predict 30 day mortality of CAP patients. Upon measuring a set of miRNAs by PCR, they conclude that high levels of miR-16-5p and miR-146a-5p are associated with lower 30-day mortality.**

**Major Concerns:**

**While the authors perform thorough statistics and take the effect of confounding variables into account, they do not address the most problematic mathematical issue, which is that the dataset is hugely imbalanced. miRNAs were measured from 106 survivors vs. 11 non survivors. This has major implications for the results interpretation, as e.g. ROC curves are inappropriate to use in such a setting. A way to deal with this would be upsampling or downsampling. But given the minuscule effect size as shown in Figure 1, I doubt that the results will hold up to this.**

**We have carefully read your concern and we would like to explain this point better.**

**Multivariate models can be used for prediction in several types of studies such as different types of cohorts, case-control designs... In the case of cohort studies, the same number of exposed vs. unexposed patients does not usually occur; it rather depends on the prevalence of the disease studied. Prediction models give us the estimate of the probability of y = 1 (Pr (ŷ = 1). This estimate is obtained from the multivariate logistic regression model followed by the estimation of the area under the curve (AUC) for a given Se (curve sensitivity). We agree that in our study the proportion of exposed may be small, and therefore the estimate of (Pr (ŷ = 1) may be somewhat biased for this reason. However, we believe that in this case this bias might not be due to the imbalance of samples, since it is not a case-control study (and this can be adjusted with Se). Regarding the balance of samples, we believe that it is adequate for a prospective observational study such as this, since the mortality of the sample is similar to the mortality of the pathology, which has been described to be around 10% at 30 days in recent series** [Shindo Y, Ito R, Kobayashi D, et al. Risk factors for 30-day mortality in patients with pneumonia who receive appropriate initial antibiotics: an observational cohort study. Lancet Infect Dis. 2015; 15 (9): 1055-1065. doi: 10.1016 / S1473-3099 (15) 00151-6]. **Furthermore, even though the sample may seem small, we have made an estimate of the power of the sample size and have obtained a good value (β = 0.77).**

**Also, the authors themselves label the dots in Figure 1 as “outliers”. I have serious reservations that without these “outliers”, the data would still be significant.**

**By consensus, outliers are defined as those below the 5th percentile and above the 95th percentile. The dots in Figure 1 fulfilled these criteria¸ that is why we labelled them as outliers. Regarding the significance of results without these outliers, we have eliminated them from the sample, and we have repeated the analysis of association of both miRNAs with mortality at 30 days, using unequal variance Student's t-test. After this analysis results maintain statistical significance. Both the process and the result can be seen below.**

```
First: hsamiR165p_NUM
Second: hsamiR146a_NUM
EXITUS_30D |       p50       p25       p75       p95        p5
-----------+--------------------------------------------------
        No |    34.067  22.48912  75.80192  128.5337  13.92374
           |  .3816975  .0959101  1.195259  4.310969  .0229395
-----------+--------------------------------------------------
       Yes |  30.47639  26.17498  49.37979   59.6065  16.28638
           |   .309985  .0546705  .3427123   .410152  .0246203
-----------+--------------------------------------------------
     Total |  33.73551  22.52498  66.32407  128.5337  13.92374
           |  .3362944  .0959101  1.119983  4.310969  .0229395

Two-sample t test with unequal variances   -    hsamiR165p_NUM
------------------------------------------------------------------------------
   Group |     Obs        Mean    Std. Err.   Std. Dev.   [95% Conf. Interval]
---------+--------------------------------------------------------------------
      No |      95    48.41939    3.475414    33.87414    41.51888    55.31991
      Yes |      11    35.22635    4.496141    14.91201    25.20833    45.24438
---------+--------------------------------------------------------------------
    diff |            13.19304    5.682762                1.479174    24.90691
------------------------------------------------------------------------------
P= 0.0288
Two-sample t test with unequal variances     -     Second: hsamiR146a_NUM
------------------------------------------------------------------------------
   Group |     Obs        Mean    Std. Err.   Std. Dev.   [95% Conf. Interval]
---------+--------------------------------------------------------------------
      No |      96    .8059193    .1026217    1.005483    .6021894    1.009649
      Yes |      11    .2331101    .0435301    .1443731    .1361189    .3301012
---------+--------------------------------------------------------------------
    diff |            .5728092    .1114724                 .351682    .7939364
------------------------------------------------------------------------------
P= 0.0000
```

**Detecting miroRNAs in plasma by PCR is an error-prone method.**

**Although some works recommend the use of serum or other biological fluids for the determination of miRNAs as biomarkers, this question is still unresolved.**

**In fact, several comparative studies in healthy patients recommend the use of plasma as sample of choice to measure circulating miRNAs, since it has been shown that RNA is released during clot formation in the process of obtaining serum, which could change the repertoire of circulating miRNAs** [Wang K, Yuan Y, Cho JH, et al. Comparing the MicroRNA Spectrum between Serum and Plasma. PLoS ONE 2012; 7: e41561. doi:10.1371/journal.pone.0041561]. **Some studies that question the use of plasma for this purpose suggest that the variability of the results can be mitigated with the use of spike-ins to control technical factors that affect final miRNA levels, as we do in our work** [Brunet-Vega A, Pericay C, Quílez ME, Ramírez-Lázaro MJ, Calvet X, Lario S. Variability in microRNA recovery from plasma: Comparison of five commercial kits. Anal Biochem. 2015; 488: 28-35. doi: 10.1016 / j.ab.2015.07.018]. **Furthermore, many of the works on miRNAs in sepsis have used plasma for their determination** [Wu X, Yang J, Yu L, et al. Plasma miRNA-223 correlates with risk, inflammatory markers as well as prognosis in sepsis patients. Medicine (Baltimore) 2018; 97: e11352. doi: 10.1097 / MD.0000000000011352] [Caserta S, Kern F, Cohen J, et al. Circulating Plasma microRNAs can differentiate Human Sepsis and Systemic Inflammatory Response Syndrome (SIRS). Sci Rep 2016; 6: 28006. doi: 10.1038 / srep28006].

**Taking all together, we chose whole plasma as the biological sample to carry out the determination of circulating miRNAs in this work.**

**The authors should provide detailed information (see MIQE guidelines for reference) on the detection process.**

**We have added extra information in the Material and Methods section about the detection process following MIQE guidelines (essential information).**

**Minor Concerns:**

**The study is generally well written, but some errors remain, e.g.**

**Line 102: procalciton should be procalcitonin**

**Line 281: Typo in “interestingly”**

**Both errors have been corrected**

**Line 285: I would refrain from using the word “evolution” in this manuscript´s context
"Evolution" has been substituted by other terms (such "trends" or "progression") where appropriate.**

**Figure Legends need more information and axis labels need to show the type of data presented (not just "miR-146a")**

**More information has been added to figure legends in order to make them clearer. Axis labels in *Figure 1* have been changed to *Relative expression levels of miR-146a* and *Relative expression levels of miR-16-5p*.**

**Reviewer #3: This study showed high admission levels of miR-146a and miR-16-5p were significant predictors of low 30-day mortality in community acquired pneumonia (CAP). It could be a useful biomarker for prognosis of CAP. I have some major comments for improving the paper.**

**1. Materials and methods**

**- Is there any information about study period and how many patients were included, excluded and the reasons why they were excluded in this prospective study?**

**Most of the information about study period and initially included patients was already included in the first paragraph of Material and Methods. We have added the word “consecutive” to further clarify patient recruitment.**

**The paragraph is now changed as follows *"Prospective observational study in a cohort of 153 consecutive patients admitted for CAP in 2015 at a university hospital in Spain. Patients older than 18 years diagnosed with CAP in the Emergency Room were included in the study".* The inclusion criterion “signing the informed consent” is now detailed in the Ethical Principles section.**

**Regarding exclusion criteria, a flowchart of the detailed technical criteria for exclusion of patients has been added in Supplementary Data as Figure 1.**

**2. Results**

**-why the variables of hypercholesterolemia, stroke, cognitive impairment were not included in multivariate model as they were statistical significant in univariate analysis?**

**All these possible confounding factors (variables with p ≤ 0.10 in the univariate analysis) were included in the multivariate model, as well as other variables described as potentially significant in all previous published studies in patients with CAP (e.g. sex).**

**With these variables we created a multivariate model that was already statistically valid (saturated model). However, we decided to progressively optimize the model until obtaining a model with a reduced number of variables (parsimonious model), showing a good goodness of fit, measured through the likehood ratio. Our final adjusted multivariate model was the 5-variable model shown in the manuscript.**

**To illustrate the aforementioned process, the initial hierarchical model that led to the proposed final adjusted multivariate model is shown below.**

```
------------------------------------------------------------------------------------
          EXITUS_30D | Odds Ratio   Std. Err.      z    P>|z|     [95% Conf. Interval]
---------------------+----------------------------------------------------------------
                 age |   1.243394   .1106221     2.45   0.014      1.04443    1.480262
           co_dyslip |   28.49155   40.01162     2.39   0.017     1.816963    446.7722
           co_stroke |   .2517313   .2803734    -1.24   0.216     .0283717    2.233516
   modCHARLSON_index |   1.166904   .2040374     0.88   0.377     .8283228    1.643883
      SCORE-OP_index |   3.66e+15   8.81e+16     1.49   0.137     .0000116    1.15e+36
   institucionalized |   1.031316   1.316902     0.02   0.981     .0844269    12.59804
         co_dementia |   1.911393   2.180035     0.57   0.570     .2044154    17.87253
    broncoaspiration |   32.40306   55.28328     2.04   0.041     1.143773    917.9775
        sudden_onset |   2.607586   3.738713     0.67   0.504      .156964    43.31888
      pleuritis_pain |   .1445987   .3154205    -0.89   0.375     .0020109    10.39761
                 SBP |     1.0323   .0331758     0.99   0.323     .9692822    1.099415
                  HR |   1.028183   .0186859     1.53   0.126     .9922037    1.065466
             ADM_HCT |   .9348093   .0798333    -0.79   0.430     .7907331    1.105137
     ADM_Neutrophils |   .9739891    .122632    -0.21   0.834     .7609952    1.246598
     ADM_Lymphocytes |   .9079161   .1801299    -0.49   0.626     .6154148    1.339441
           ADM_Album |    .405816   .4442956    -0.82   0.410     .0474697      3.4693
         ADM_chTOTAL |   .9777422   .0188828    -1.17   0.244     .9414243    1.015461
             ADM_BUN |   .9919923   .0393185    -0.20   0.839     .9178467    1.072128
          ADM_proTot |   1.093898   .7972458     0.12   0.902     .2621869    4.563968
      alt_level_cons |   8.38e-10   1.30e-08    -1.34   0.179     4.77e-23    14712.04
          exitus_30d | Odds Ratio   Std. Err.      z    P>|z|     [95% Conf. Interval]
---------------------+----------------------------------------------------------------
```

**-the high standard deviation of miR-146a,miR-16-5a in alive group(table 2) indicates that the data points are spread out over a large range of values, some alive patients may have values of miR-146a,miR-16-5 within the range of the deceased group, which may not distinguish between them and what is the percentage. .**

**To clarify this point, we have represented the distribution of expression values of miRNAs of interest in the live a dead patient groups using a cloud-plot.**

**As shown below, many of the alive patients have values of both miRNAs above the level of deceased patients, with only a few being outliers. Specifically, patients with relative amounts of miR-146a above 2 FC are clearly outliers, while the rest of the patients appear to belong to the same group distribution. Similarly, patients with relative amounts of miR-16-5p above 150 FC are outliers, while the rest of the patients are not.**

**This distribution conditions two facts: that from a certain level of miR-16-5p and miR-146a we did not find any deceased patients - which could be interpreted as very protective - and that the statistical comparison of their means for unequal variances shows a statistically significant association.**

**-furthermore, the value maybe also unsteady to reflect a reliable biomarkers because of possible fluctuation or embedded extreme level in alived group . Do you have the information about the statistical analysis of the median or the trend of serial level of miR-146a,miR-16-5a between two groups, which may avoid one-point bias and became more reliable.**

**We understand well the reviewer's assessment of the difference in the dispersion between live and dead patients, and possible concern about extreme values. Therefore, we have performed the same analysis of both miRNAs, excluding outliers (values below P5 and above P95, according to the usual definition). Student T-test comparisons for unequal variances maintain statistical significance.**

```
First: hsamiR165p_NUM

Second: hsamiR146a_NUM

EXITUS_30D |       p50       p25       p75       p95        p5
-----------+--------------------------------------------------
        No |    34.067  22.48912  75.80192  128.5337  13.92374
           |  .3816975  .0959101  1.195259  4.310969  .0229395
-----------+--------------------------------------------------
       Yes |  30.47639  26.17498  49.37979   59.6065  16.28638
           |   .309985  .0546705  .3427123   .410152  .0246203
-----------+--------------------------------------------------
     Total |  33.73551  22.52498  66.32407  128.5337  13.92374
           |  .3362944  .0959101  1.119983  4.310969  .0229395


Two-sample t test with unequal variances   -    hsamiR165p_NUM
------------------------------------------------------------------------------
   Group |     Obs        Mean    Std. Err.   Std. Dev.   [95% Conf. Interval]
---------+--------------------------------------------------------------------
      No |      95    48.41939    3.475414    33.87414    41.51888    55.31991
     Yes |      11    35.22635    4.496141    14.91201    25.20833    45.24438
---------+--------------------------------------------------------------------
    diff |            13.19304    5.682762                1.479174    24.90691
------------------------------------------------------------------------------
P= 0.0288


Two-sample t test with unequal variances     -     Second: hsamiR146a_NUM
------------------------------------------------------------------------------
   Group |     Obs        Mean    Std. Err.   Std. Dev.   [95% Conf. Interval]
---------+--------------------------------------------------------------------
      No |      96    .8059193    .1026217    1.005483    .6021894    1.009649
     Yes |      11    .2331101    .0435301    .1443731    .1361189    .3301012
---------+--------------------------------------------------------------------
    diff |            .5728092    .1114724                 .351682    .7939364
------------------------------------------------------------------------------
P= 0.0000
```

**-Could you give the cutoff value of miRNAs in AUC model with only miRNA for predicting the 30-day mortality.**

**Responding to the reviewer's request, we show ROC curve with every single significant miRNA, separately.**

**However, we think that we should not include these graphs in the text for various reasons. First, and most important, the determination of miRNAs is measured in relative expression, in relation to exogenous and / or endogenous normalizers; therefore, although the trends may (and should) be reproducible by other researchers, the numerical value may not be so. Second, we believe that it is not correct to offer cut-offs in studies not validated in other cohorts. Finally, in this case the ROC curve is constructed from the logit of the model (nothing to do with its numerical value), so the cut-off would not have a simple direct interpretation.**
