# Supplementary material for: Usefulness of circulating microRNAs miR-146a and miR-16-5p as prognostic biomarkers in community-acquired pneumonia
Source: PLoS One. 2020 Oct 23;15(10):e0240926. doi: 10.1371/journal.pone.0240926 (PMC7584179; doi:10.1371/journal.pone.0240926)
Supplement: Attachment — Submitted filename: Response_to_Reviewers_22_09_20.docx [file pone.0240926.s003.docx]

**PONE-D-20-09507
Usefulness of circulating microRNAs miR-146a and miR-16-5p as prognostic biomarkers in community-acquired pneumonia**
-----------

***Response to Reviewers***

All questions and suggestions raised by the reviewers are shown marked **in blue**, and our replies are marked **in black**, in order to facilitate a better understanding.

**Reviewer #1:** I want to thank the authors for addressing all my raised issues. Therefore, I have no more relevant concerns.

We greatly appreciate your entire review process and your valuable contributions to improve the manuscript.

However, I would like to encourage the authors once again to consider the opportunity of reclassification indices, e.g. NRI + IDI (as already proposed in R1). The authors themselves have pointed out that their data is prone to substantial biases needing further validation. Thus, a likely overfitted statistic along with less robust data (as also noted by Reviewer 2) casts doubt on the validity of their results. Therefore, reclassification indices can certainly add value here.

We have followed Reviewer 1's request and we have calculated the net reclassification index (NRI) and the integrated discrimination index (IDI). The NRI estimated for our multivariate model vs CURB-65 was 59.61% (SE = 1391.69; p = 1) and vs PSI was 39.54% (SE = 1029.78; p = 1). The interpretation of these results could be that our multivariate model (age, history of aspiration, miR-146a and miR-16-5p) reclassifies patients 40% / 60% better than these classic scales, although results are not statistically significant.

On the other hand, the IDI estimate for our model vs. CURB-65 was -0.40 (SE = 0.12; p = 0.007) and vs. PSI was -0.41 (SE = 0.11; p = 0.004), whose interpretation could that our model reclassifies patients worse than these classic prognostic scales, although with a minimal magnitude of change.

Therefore, we believe that the correct interpretation of these indices, together with the comparison of the AUCs, previously presented in the manuscript, is that our model including both miRNAs is at least as good as the CURB-65 and PSI scores, and therefore useful for prognosis prediction in patients hospitalized for CAP.

To include this new requested approach, we have modified the Statistical Analysis subsection, within the Material and methods section:

*In addition, the net reclassification index (NRI) and the integrated discrimination index (IDI) were calculated.*

We have also modified the Results section:

*In addition, we sought to test how the model proposed could classify patients according to 30-day mortality compared to classic prognosis scales such as CURB-65 and PSI. For that purpose, NRI and IDI were assessed. The NRI estimated for our multivariate model vs CURB-65 was 59.61% (SE=1391.69; p=1) and vs PSI was 39.54% (SE=1029.78; p=1); IDI estimation for our model vs. CURB-65 was -0.40 (SE=0.12; p=0.007) and vs. PSI was -0.41 (SE=0.11; p=0.004).*

Lastly, we have made minor changes in the Discussion section.

*In our sample of patients admitted with CAP, this adjusted model was at least as good at predicting mortality at 30 days as the classic CURB-65 and PSI prognostic scales, after comparison of AUCs and evaluation of reclassification indices NRI and IDI.*

**Reviewer #2:** Thank you for considering the points I raised.
I believe that, as the authors show with their statistical approach, that there are, in retrospective analysis, significant differences between the mortality groups. I do not believe that these differences have prognostic value, because the overlap between the miRNA levels of the different mortality groups is very high. On an individual patient basis, measuring these miRNAs will allow no mortality prognosis.
The model the authors derive might have its merits, though.

We respectfully disagree with the observation "on an individual patient basis, measuring these miRNAs will allow no mortality prognosis". According to our data, all patients with miR-16-5p levels greater than 60 FCs survive until follow-up, so having high levels of this miRNA is a very clear protective marker. Similarly, levels greater than 1 FC of miR-146a are equally protective. Although the number of patients in this group is small, a good prognosis could be estimated for individual patients with high levels of this miRNAs, without considering other factors.

The determination of a cut-off point with value far from the mean/median that reliably predicts a good evolution of CAP could be used in the future within broad prognostic scales or even within therapeutic decision trees. Therefore, we consider that values for these miRNAs could be useful in the future for individual patients, although we recognize that direct clinical translation is not possible at this time, as the models needs to be validated and standardized.

In this regard, we already underlined this point in the discussion with the phrase: *Nevertheless, the prognostic value of miR-16-5p and miR-146a described in this work needs to be further confirmed in routine clinical practice* (lines 351 -352).

One thing I only noticed when reading the revised manuscript: Was the spike-in done before or after RNA purification? It reads as if it was done afterwards, and this would mean it does not reflect changes in RNA composition that are introduced during the purification procedure. If this is the case, changes in abundance might be artefacts.

Thank you for the comment. After revising the information about miRNA purification in the Materials and Methods section we realized that the description of spike-in addition is somewhat ambiguous, and it may lead to this interpretation. The addition of spike-in as internal controls was carried out sequentially, as proposed in the user guidelines of the RNA purification kit, and as established as good standard practice.

According to this, the spike-ins UniSp2, UniSp4 and UniSp5 were added before the column RNA extraction process and UniSp6 was added prior to retrotranscription to cDNA.

To better clarify this point, the following sentence in the Materials and Methods section has been modified:

[…] *synthetic RNA controls were added in this process (spike-ins UniSP2, UniSp4 and UniSp5 before RNA extraction, and UniSP6 before retrotranscription to cDNA).*

Importantly, apart from citing literature and some remarks in the methods section, the authors did not provide, as I requested, data on the detection quality of the microRNAs. This plus the fact that the link to the DOI 10.5281/zenodo.3930832 returns "DOI not found" makes it still impossible to evaluate the results.

Regarding the DOI address of the repository with the original data, we made a mistake including a wrong DOI, for which I would like to apologize on behalf of the entire research team. The reason for the error was that the data access address during the uploading process to zenodo.org is different from the definitive address after data publication. We were not aware of this change and, unfortunately, we did not verify data availability once published. The correct and verified address is <https://doi.org/10.5281/zenodo.3930832>

We have corrected this address both in the manuscript and in the information for submission.

Data on the quality of microRNA detection is provided in detail in the aforementioned repository. As explained in the text, all samples were subjected to an external control using PCR control plates (*QC control Panel*, Exiqon), using only those samples that had strictly passed all quality controls for the rest of the study, as detailed in Supplementary Figure 1.

We would like to apologize again for providing an incorrect web access address, which has prevented reviewer 2 from reviewing these data.

**Reviewer #3**: The authors have done additional great works and providing data to adequately addressed response to the comments.

We greatly appreciate your entire review process, in addition to your valuable contributions to improve the manuscript.
